# Potential Role of Mosses in Evaluating Airborne Microplastic Deposition in Terrestrial Ecosystems

**DOI:** 10.3390/jox16010021

**Published:** 2026-01-24

**Authors:** Roberto Bargagli, Emilia Rota

**Affiliations:** Department of Physics, Earth and Environmental Sciences, University of Siena, Via P.A. Mattioli 4, IT-53100 Siena, Italy; roberto.bargagli@unisi.it

**Keywords:** airborne microplastics, biomonitoring, bioaccumulation, future research, mosses, MP interception, MP retention

## Abstract

The deposition of airborne microplastics (MPs) poses potential risks to human health and terrestrial ecosystems. Therefore, suitable mitigation efforts are needed, as is knowledge of their deposition patterns in inhabited and remote regions. Currently, there are no standardized protocols for monitoring airborne MPs, and implementing and managing automatic monitoring systems would be costly and feasible only in a few fixed locations. Over the past few decades, several species of cryptogams have proven to be reliable biomonitors of persistent atmospheric contaminants. Due to the lack of standardized methodologies, the results of preliminary biomonitoring surveys for MPs have been inconsistent and difficult to compare. However, they clearly indicate higher MP concentrations in epigeic mosses than in epiphytic lichens (collected at the same site or experimentally exposed in parallel in bags). This review discusses the morphophysiological features that favor the entrapment and retention of intercepted MPs in mosses, as well as the field and laboratory activities necessary to determine whether these organisms progressively accumulate airborne MPs as a function of the exposure time. Steps for future research needed to develop a cost-effective, reliable and easily applicable biomonitoring methodology are suggested. Evaluating the advantages of active moss biomonitoring over sampling atmospheric bulk deposition or exposing suitable commercial materials is recommended.

## 1. Introduction

Global production of plastics doubled in the first two decades of this century, and forecasting models suggest that, in a business-as-usual scenario, it will exceed 700 million tons by 2040 [1]. Currently, less than 10% of plastic waste is successfully recycled; the rest is incinerated, ends up in landfills, or is released directly into aquatic and terrestrial ecosystems. Synthetic polymers are also leaked into the environment during their production and when used for packaging, textiles, constructions and many other purposes. In terrestrial and aquatic environments, large plastic litter slowly degrades and fragments into small particles of microplastics (MPs: 0.001–5 mm) and nanoplastics (NPs: <1 µm) through solar radiation, weathering processes, and microbial activity [2,3,4]. Thus, even if the production of synthetic polymers decreases and/or recycling rates increase in the near future, the amount of MPs and NPs in the environment will remain significant due to the fragmentation of larger items already present in soil, water and sediments [2]. Furthermore, very small plastic particles are intentionally manufactured for various applications, such as textiles, paints, cosmetics and personal care products. Others are produced through the wear of car tires. Due to their persistence and small size, a variety of MPs, mainly in the form of fibers and fragments, have become pervasive and ubiquitous global contaminants through atmospheric and aquatic transport [3,4,5].

In the environment, MPs can easily be ingested by aquatic and terrestrial organisms, including humans, through breathing, eating or drinking. Ingestion of MPs can pose health risks through abrasion and other physical damage to internal organs, leaching of chemical additives used to improve the performance of plastic products, and introduction of other persistent contaminants and microorganisms adsorbed onto their surfaces [6,7]. However, knowledge of their potential negative biological or ecological effects is incomplete, and commitments to managing and reducing plastic waste, as well as researching their distribution, bioavailability, and biological effects in aquatic and terrestrial ecosystems, are urgently needed [1].

Every year, about 8.9 million tons of plastic are released onto land, which is about double the amount leaked into the oceans (3.8 million tons) [8]. Moreover, due to exposure to greater temperature excursions and UV radiation, plastic waste in terrestrial environments degrades more easily into small particles than in water bodies [9]. In soils, the reduction in size and weathering of MPs increase their bioavailability, and the absorption of additives and adsorbed contaminants can affect the structure and functionality of microbial communities [10]. Generally, MPs absorbed by plant roots are slowly translocated to aboveground tissues; however, Li et al. [11] recently highlighted the absorption of MPs through the stomata of maize leaves and their subsequent translocation to vascular tissues via the apoplastic pathway (i.e., the intercellular space). Thus, terrestrial organisms are directly exposed to airborne MPs through inhalation and ingestion via drinking water and food of animal and plant origin. In humans, intake is further increased by processing, handling and packaging food and beverages [12]. In order to address the growing challenges posed by MPs, it is necessary to establish regulatory frameworks and develop environmental monitoring networks [4]. However, the multiple sources of airborne MPs and the effects of meteorological conditions on their resuspension, atmospheric transport and deposition [13] make it difficult to assess spatial and temporal changes in their deposition patterns with conventional monitoring devices. Detecting atmospheric MP concentrations (items m^−3^ of air) with active sampling devices is expensive, requires electricity, and only provides data from a few sites. As with other persistent atmospheric contaminants, developing standardized, cost-effective biomonitoring methods could provide useful complementary information to the quantitative data obtained with monitoring devices, allowing for MP deposition maps to be traced over large and remote areas [14].

Preliminary biomonitoring surveys using lichens and mosses suggest that these organisms accumulate higher MP concentrations in urban and landfill areas than in control areas (e.g., [15,16,17,18]). However, due in part to the lack of standardized methodologies, the results of these studies are highly variable and difficult to compare (e.g., [19,20,21]). It is often assumed that MPs intercepted by exposed organisms are progressively accumulated like other persistent atmospheric contaminants. However, some field and laboratory studies indicate that not all plastic fragments are retained (e.g., [19,21,22]). Weather conditions and other factors probably prevent cryptogams from reliably providing semi-quantitative data on spatial and temporal changes in the bulk atmospheric deposition of MPs. Nevertheless, while these organisms likely do not behave as true biomonitors, the plastic microfibers (MFs) and fragments retained in their thalli can provide information on sources and deposition [23].

In a previous review [23], we discussed possible interactions between MPs and the thalli of epiphytic lichens. Based on the results of biomonitoring surveys, we concluded that these organisms, like the leaves of plants, are not very efficient at retaining intercepted particles and cannot be used as reliable biomonitors. In comparison, bryophytes have a morphology that is better suited for trapping intercepted particles, and moss carpets in terrestrial ecosystems are entirely exposed to wet, dry, and occult atmospheric deposition. Comparative studies on the presence of MPs in cryptogams collected from the same field site or exposed in parallel show a higher number of MPs in mosses than in lichens [18,23]. Therefore, when searching for useful cryptogam species to be used as potential biomonitors of airborne MPs, it seems appropriate to evaluate the opportunities offered by mosses. To this end, the present review analyses the available MP biomonitoring results and suggests methods to determine whether mosses progressively accumulate intercepted MPs. The research steps needed to develop a reliable, cost-effective, and widely applicable biomonitoring methodology are also outlined.

## 2. Mosses as Bioindicators and Bioaccumulators of Persistent Atmospheric Contaminants

The empirical use of plants to evaluate soil quality for agricultural and metal exploitation purposes began in China around four thousand years ago. In the second half of the 16th century, the first reports describing plant species as indicators of mineral deposits appeared in Europe [24]. This knowledge formed the basis for the development of modern scientific geobotanical methods for mineral prospecting. Beside this, the combustion of huge quantities of fossil fuels that began during the Industrial Revolution caused atmospheric pollution and acid precipitation, posing a critical hazard for terrestrial and freshwater ecosystems and human health. Since some gaseous and particulate atmospheric pollutants caused easily identifiable damage to plant leaves and cryptogams, many species of these organisms became useful bioindicators of atmospheric pollution [14]. However, following the introduction of regulatory and technological measures in many countries to reduce atmospheric concentrations of pollutants such as sulfur dioxide (SO_2_), hydrogen fluoride (HF), and lead (Pb), as well as acid precipitation, the role of plants as bioindicators of air quality has significantly decreased. Currently, except for tropospheric ozone (O_3_) and nitrogen (N) compounds, the concentrations of most phytotoxic atmospheric contaminants (whether legacy or of emerging interest) are generally low and unlikely to cause easily detectable biological effects on plants. Moreover, due to their hydrophobic nature, ad/absorbed MPs should have scarce chemical interactions with the metabolism of cryptogams and are unlikely to cause visible injuries or symptoms. Nevertheless, given their wide distribution in all terrestrial ecosystems, long life cycles and suitable surfaces for the ad/absorption of gaseous, soluble and particulate constituents of atmospheric deposition, mosses could play an important role as bioaccumulators of airborne MPs.

The use of mosses as accumulative biomonitors began in the late 1960s, when the “European Moss Survey” was launched by Swedish and Norwegian universities (on behalf of the Nordic Council of Ministers) to track the large-scale atmospheric deposition of heavy metals [14]. The coordination was then transferred to the International Cooperative Programme on the “Effects of Air Pollution on Natural Vegetation and Crops” (ICP Vegetation), within the framework of the Convention on Long-Range Transboundary Air Pollution (LRTAP) [25]. Over the years, large-scale biomonitoring of trace metals has been extended to include other contaminants and experimental protocols have been implemented. This type of monitoring is now performed regularly every five years in several countries. In 2020, MPs were added to the list of pollutants, along with methods for sampling and analyzing moss samples [25].

Using mosses collected in the field or exposed in bags as bioaccumulators of contaminants is a cost-effective approach that provides valuable information on the sources and spatiotemporal patterns of atmospheric contaminant deposition. Passive biomonitoring is essential in remote regions where logistical constraints render conventional monitoring devices impractical [26]. Although quantitative data from biomonitoring surveys can provide useful information that complements that recorded in monitoring stations, it generally cannot be used to reliably estimate the bulk atmospheric deposition of contaminants (e.g., [26,27]). Cryptogams intercept atmospheric contaminants through processes that are more complex and different from those of conventional atmospheric deposition samplers. In addition, as Varela et al. [28] recently discussed, most biomonitoring surveys focus too much on the uptake of water, nutrients, and contaminants from the atmosphere, ignoring the interactions and exchanges between cryptogams and the substrate. Samples of epigeic and epilithic mosses collected in the field for passive biomonitoring are not washed prior to analysis, and a significant proportion of their total element concentrations are often due to entrapped soil and rock dust particles. Therefore, to evaluate the actual contribution of elements from atmospheric deposition, it is necessary to subtract the amount attributable to soil contamination from the total measured concentrations of elements [29]. In the case of MPs, it seems unlikely that their concentrations in bryophytes can reflect those in bulk atmospheric deposition. This is because mosses probably cannot retain all intercepted particles, and some could also come from local resuspension due to winds or raindrops.

## 3. Microplastic Concentrations in Native and Exposed Mosses

For this study, a systematic review was conducted to retrieve peer-reviewed articles from the PubMed, Google Scholar and ScienceDirect databases using the keywords “Exposure”, “Interception”, “Presence”, “Detection”, “Uptake”, “Accumulation”, AND “Microplastics”, AND “Mosses”. The first biomonitoring of MPs with terrestrial mosses was performed five years ago [15] using the glittering woodmoss *Hylocomium splendens* [15]. Since then, various species of Sphagnaceae, Hylocomiaceae, and Hypnaceae have been employed as active and passive bioaccumulators in urban and control areas (Table 1). Before analyzing the results obtained thus far, it is important to note that, although a protocol was drafted for moss biomonitoring [25], there are no reliable, standardized procedures for quantifying and characterizing MPs in all environmental matrices. The variety of species and methodologies used and the possible effects of local meteorological conditions on the retention of intercepted MPs make comparisons among the data summarized in Table 1 inconclusive. Some publications do not indicate the smallest size of analyzed particles, and among those that do, the values vary from 30 to 300 µm. Comparisons should be more accurate between mosses exposed in bags for the same period of time. For instance, in samples exposed in urban and rural areas in Southern Italy for six weeks [18], MP concentrations (smallest size: 200 µm) were about one order of magnitude higher than those in moss bags exposed for a similar period in southern Ontario (Canada) [17] (Table 1). Note, however, that the latter study area had traffic intensity of up to 450,000 vehicles per day and analyzed MPs as small as 30 µm [17] (Table 1).

In samples of *Pseudoscleropodium purum* collected in remote areas of central Italy at elevations ranging from 440 to 1140 m a.s.l., Jafarova et al. [31] measured MP concentrations about double those reported in their more recent study [33], which was also carried out in semi-natural sites of central Italy using the same methodologies (Table 1). In the latter study [33], the moss biomonitoring protocol [25] was applied to collect samples of different species (mainly *Hypnum cupressiforme*) from 33 sites randomly selected within 50 km × 50 km grids in Tuscany, at elevations ranging from 46 to 1079 m a.s.l. To our knowledge, this is the first large-scale biomonitoring study using mosses. Despite the methodological uncertainties and a lack of knowledge regarding the actual capacity of cryptogams to intercept and retain MPs, the results provide information on the spatial deposition of MPs at a regional scale. As observed in samples of bulk atmospheric deposition [3,13], the concentration of MPs in moss increased in areas with higher population densities (within a 10 km buffer), suggesting that in Tuscany local sources of MPs are likely more important than long-range atmospheric transport [33]. Textile fibers and polyethylene terephthalate were the dominant polymers in all samples (Table 1), and the occurrence of certain polymers indicated that local agricultural activities also contributed to environmental contamination by MPs [33]. Although it is commonly believed that rainfall plays a pivotal role in the transport and deposition of MPs [13], in Tuscany the concentrations of MPs in moss were negatively associated with rainfall amounts. Therefore, dry deposition was presumed to be prevalent [33].

Our knowledge of the atmospheric transport and deposition of MPs under different meteorological conditions is limited. An analysis of 24 studies performed in 15 different countries revealed that MP deposition rates can vary by five orders of magnitude, and hot weather and wind turbulence contribute to higher values in arid and tropical regions [35].

Interestingly, statistically significant correlations were found between MP concentrations and those of certain lithophilic elements, such as chromium (Cr) and nickel (Ni), in moss biomonitoring throughout Tuscany [33]. Suspended soil and rock dust particles are efficiently trapped in epigeic mosses, influencing the distribution of total lithophilic element concentrations [29]. Therefore, it cannot be ruled out that epigeic mosses can intercept and accumulate plastic particles that are re-suspended by wind or raindrops.

## 4. Features Making Mosses Potential Biomonitors of Microplastics

Biomonitoring allows one to map the atmospheric deposition of persistent contaminants over large and remote areas without the need for repeated sampling within short time intervals. This approach is also cost-effective because heavy metals, persistent organic pollutants, and artificial radionuclides accumulate in cryptogams at much higher concentrations than in atmospheric deposition samplers. Therefore, they can be determined using more accessible instrumentation, with less risk of sample contamination during collection and analysis. Conversely, due to their re-suspension and atmospheric transport, MPs are pervasive and ubiquitous in all terrestrial ecosystems, and their concentrations in bulk or wet atmospheric deposition are usually detectable in samples collected far from urban and industrial areas [5,36]. Moreover, even though there are no standardized analytical methods, extracting and characterizing MPs from atmospheric deposition is easier than from biological samples. In indoor environments, for instance, where MP concentrations are higher than outdoors, sampling can be performed using Petri dishes or glass slides with an adhesive surface. Li et al. [37] have recently shown that MPs can be analyzed directly without sample preparation. Outdoors, the deposition rate of airborne MPs is usually determined by collecting bulk atmospheric deposition in a glass container with a funnel. This low-cost approach does not require electricity and can be easily applied over large areas. To reliably evaluate the results, the meteorological conditions during sampling should be recorded. However, perhaps meteorological measurements are not indispensable in long-term surveys. In fact, the deposition rate of MPs (detected down to a cutoff of 10 µm) in samples of bulk atmospheric deposition collected monthly in northern Germany (from August 2019 to July 2020) did not change significantly across the four seasons [38]. The mean deposition rate was two to three times higher in densely populated urban areas than in rural areas. In coniferous forests, values did not change between the dormant and vegetation periods. Interestingly, in beech or oak forests, the deposition rate was lower during the leafless period than the leaf-bearing period (36 and 70 items m^−2^ day^−1^, respectively). Broadleaf trees intercept more MPs during the vegetation period, which can be washed off by rain and contribute to collection in bulk deposition samplers [38]. These findings confirm the importance of collecting samples far from tree canopies during passive biomonitoring with moss.

Due to the limitations in microplastic extraction and analysis techniques, as well as uncertainties regarding moss effectiveness in retaining intercepted MPs, it is impossible to evaluate whether moss behaves as a real accumulative biomonitor (that is, if it progressively accumulates MPs in concentrations that reflect those in bulk atmospheric deposition to some extent). Despite these shortcomings, most moss biomonitoring surveys assume progressive bioaccumulation of MPs and estimate mean annual deposition rates (e.g., [15,18,31]). These estimates often align with the numerous data points reported in the literature on MP concentrations in atmospheric deposition. However, these estimates are unreliable because samples of cryptogams and atmospheric deposition were collected at different sites and times [23]. Moreover, MP concentrations in atmospheric deposition are highly variable due to changes in meteorological conditions, population density and activities, and the physical properties of plastic particles [13,35].

The assumption that mosses are among the most suitable organisms for MP biomonitoring essentially stems from the results of comparative studies showing that, for an equal weight, mosses have a higher content and variety of MP shapes and sizes than lichens collected from the same site [31,34] or exposed in parallel [18]. In particular, the latter study found that moss bags were more efficient at retaining the smallest anthropogenic microfibers, and that MP concentrations in mosses increased consistently during the 6-week exposure. In contrast, a loss was detected in some lichen bags. These results are not surprising as previous passive biomonitoring surveys have shown mosses to be more effective than lichens in accumulating particulate trace elements [39,40].

**Figure 1:**
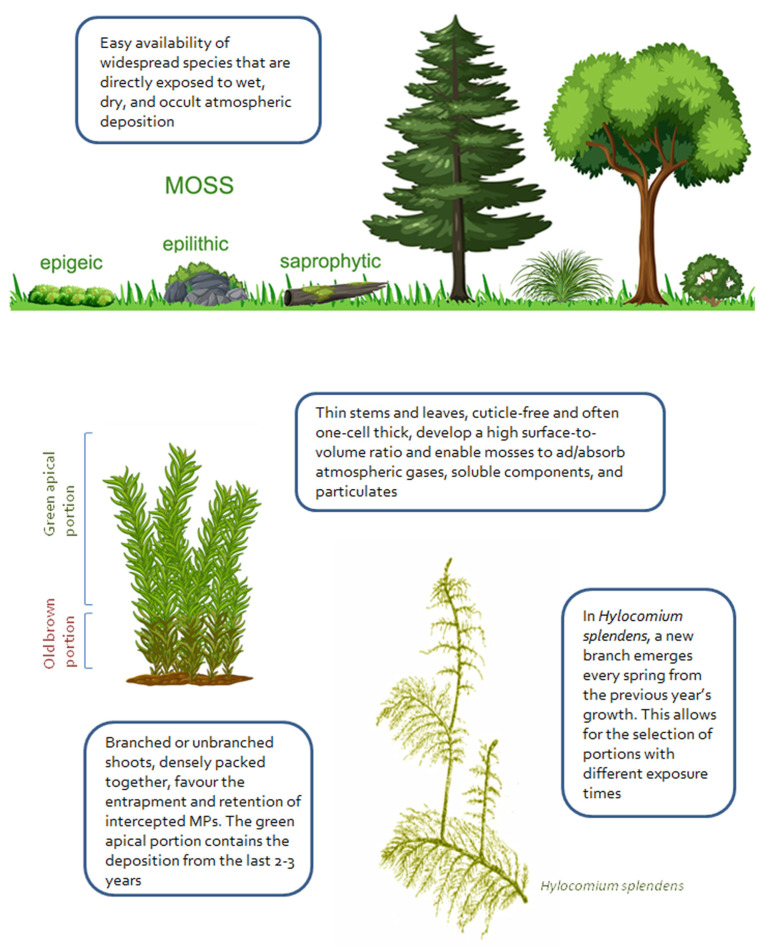
Moss features that could contribute to the retention of intercepted microplastics.

The physical–chemical processes that influence the uptake and retention of MPs in cryptogams are unknown. The different chemical compositions of moss cell walls and lichen tissues probably play a minor role. Unlike positively charged metal ions, which can bind to negatively charged sites on moss cell walls and plasma membranes, persistent, hydrophobic plastic particles likely have scarce chemical interactions with moss surfaces. Laboratory experiments with the aquatic moss *Sphagnum palustre*, for instance, showed that the retention of polystyrene nanoparticles significantly increased in devitalized mosses [41]. Thus, as demonstrated for plant leaves [21,42] and lichens [22], most plastic microfibers and fragments are simply adsorbed onto moss tissues, being thus exposed to the effects of wind and rain. The superior ability of mosses to retain MPs is likely explained by their complex 3D morphology. The green gametophyte, which is analyzed in biomonitoring surveys, consists of many stems (caulids) surrounded by thin, leaf-like phyllids that develop a high surface to dry mass ratio (Figure 1). Even the small spaces between leafy shoots and overlapping phyllids may favor the entrapment and retention of airborne particles. Samples of epigeic (ground-dwelling) moss species that grow far from tree canopies and are thus directly exposed to atmospheric deposition are collected for passive biomonitoring of atmospheric contaminants. Under these circumstances, airborne MPs that have been intercepted by green shoots may tend to descend into the underlying brown portion of caulids due to the action of rain. Indeed, the metabolically inactive brown tissues, which are normally not analyzed in biomonitoring studies, can contain very high concentrations of particulate lithophilic elements [40].

## 5. Research Needed to Evaluate the Potential of Mosses as MP Biomonitors

In recent years, there has been increasing interest in the bioeconomy and using renewable biological resources to create sustainable solutions for circular economic growth and environmental protection. In this context, Khalid and Singh [43] explored the potential use of mosses as a bioeconomy resource for bioenergy, air purification and the bioaccumulation of persistent contaminants. Considering the lack of standardized procedures for the instrumental monitoring of MPs and their growing threat to essential resources such as air, water, food, and human health, the availability of a reliable, easily applicable biomonitoring methodology using mosses would significantly increase their bioeconomical role. Furthermore, maps and information on MP deposition obtained with moss could also be used to select optimal locations for eventual future development of automatic monitoring networks.

Following several decades of biomonitoring air pollution with mosses, the most suitable species to use as bioaccumulators are rather well known (e.g., those in Table 1), as are the sampling protocols. However, to compare contaminant concentrations in samples of a moss species collected in different sites or at different times, it is necessary to analyze tissues of the same age (i.e., with the same exposure time). While it is impossible to determine the age of the different portions of a lichen thallus, mosses offer the opportunity to select and analyze the green apical part of shoots, which is the biomass produced in the last 2–3 years. To evaluate whether MPs are progressively accumulated by mosses, tissues of different ages could also be analyzed. In the moss *Hylocomium splendens*, for instance, the temporal patterns of MP bioaccumulation could be evaluated by analyzing shoots that grow from near the center of the previous year’s shoot separately (Figure 1). Also due to the action of rain, MP particles in green shoots are likely to slide down, and in other moss species analyzing the basal brown portion of shoots could provide information on MPs intercepted in the past. However, a more accurate approach to evaluating the retention and progressive accumulation of MPs is to analyze transplanted mosses collected after different periods of time and under different meteorological conditions. Preliminary surveys using moss bags have shown that this approach is promising [18], and recent experiments using a galvanized steel cage (“the moss cube”) appear to produce even better results [44]. In order to evaluate whether native or exposed moss preferentially retains MPs of a certain size, shape or density, the bioaccumulated MPs could be compared with those in samples of atmospheric deposition collected in parallel and identified using the same visual approach.

However, as emphasized in many recent papers (e.g., [6,19,23,45]), the urgent and challenging priority is to define standardized, easily applicable methods for extracting, visually identifying and counting MPs. This is an essential prerequisite for obtaining reliable and comparable results. Unfortunately, important steps remain to be defined, such as the minimum quantity of moss tissue to analyze, the most suitable extraction procedure, the particle size to recognize and the unit of measurement to express the results. Furthermore, the minimum number of particles to be analyzed for possible characterization of polymers using FT-IR or Raman spectroscopy should be established.

One of the main difficulties in MP research is reducing sample contamination during collection and especially in the laboratory. Strict protocols must be followed, such as those recently proposed through a 28-month monitoring survey of trace organics in a laboratory [46]. To ensure the accuracy of analytical determinations, it would also be useful to have a certified moss reference material that specifies the concentrations of different types and sizes of plastic particles.

When developing standardized, reproducible biomonitoring methods, it is worth considering that mosses are probably unable to progressively accumulate all MPs in atmospheric deposition, and that some of them may have been re-suspended from the nearby ground (i.e., moss data cannot be used to estimate mean annual deposition rates). However, they can still provide useful spatiotemporal information that could complement that recorded by active or passive monitoring devices, provided the biomonitoring method is reliable, cost-effective and applicable on a large scale.

Several novel approaches to processing environmental samples and extracting MPs and NPs, as well as techniques for their quantitative and qualitative characterization, have been proposed (e.g., [47,48]). However, biomonitoring methods must not require complex instrumentation, much time, or expertise from operators. Thus, as discussed by the working group studying the impact of plastic on marine wildlife (Global Plastic Ingestion Bioindicators, GPIB) [49], it will probably be necessary to limit stereomicroscopy analyses to easily identifiable MP particles (>50 µm in size?). As has been done for the atmospheric deposition of trace metals [50], if reliable, standardized methodologies for active moss biomonitoring are defined, it will also be appropriate to compare the concentrations of MPs accumulated by mosses with those accumulated by commercial materials exposed in parallel. In other words, active monitoring using moss only makes sense if it outperforms glass fiber filters or other materials in the interception and accumulation of MPs, as this would allow for a much easier recovery of MPs and avoid the collection of native mosses.

## 6. Conclusions

Atmospheric transport plays an important role in the global cycle of MPs and their deposition in terrestrial ecosystems affects the composition of essential resources, such as air, water, and soil. This represents a potential threat to human and ecosystem health. While the full bio-ecological impact of MPs is not well understood, there is an urgent need to mitigate their release and assess their deposition patterns in inhabited and remote terrestrial regions. Currently, unlike other persistent atmospheric contaminants, there are no standardized protocols or devices for monitoring airborne MPs. In any case, implementing and managing automatic monitoring systems would be costly and feasible only in a few fixed locations. Therefore, developing reliable, cost-effective and easily applicable biomonitoring methods would be useful for obtaining information on spatiotemporal variations in MP deposition. The availability of suitable accumulative biomonitors of airborne MPs could enable deposition maps to be traced for remote regions and provide useful information for the location of monitoring stations in urban and industrial areas. This could also complement the point-in-time or short-term data from air samplers once these are eventually installed.

However, due to the lack of standardized methodologies, the results of preliminary biomonitoring surveys with mosses and lichens are highly variable and difficult to compare. Moreover, although some field and laboratory studies indicate that, especially in lichens, plastic particles are not efficiently retained, annual atmospheric deposition rates of MPs have often been estimated from biomonitoring data.

Despite methodological shortcomings, the preliminary results of biomonitoring surveys indicate that mosses contain a greater number and variety of MPs than lichens, whether collected in the field or exposed in experiments. The more complex morphology and different exposure to atmospheric deposition are probably the main factors that favor a greater retention of MPs in mosses. Possible approaches to evaluating the progressive accumulation of MPs in native or experimentally exposed mosses have been suggested. It was emphasized that the definition of standardized and easily applicable methods for the extraction, identification and counting of MPs in moss samples is an essential prerequisite for the possible development of a reliable, cost-effective and easily applicable biomonitoring methodology. Once these methods have been defined, the amounts and types of MPs in bulk atmospheric deposition and those retained in mosses and suitable commercial materials exposed in the same location and under the same meteorological conditions will need to be compared.

## Figures and Tables

**Table 1:** Detection methods, abundance (items g^−1^ dry wt moss), % and length of anthropogenic microfibers (MFs) in different moss species collected in the field or exposed in bags.

Moss Species	Study Area, Active/Passive Biomonitoring	Detection and Characterization Methods	Items g^−1^ Dry wt Moss	MFs % and Size (mm)	Ref.
*Hylocomium splendens*	Remote areas (Ireland) Field sampling	Stereomicroscopy Raman spectroscopy	Range = 8–34 Mean = 24	MFs, 13–27% of which MPs, Mean length = 1.02	[15]
*Pleurozium schreberi*	Urban-to-rural gradient (Canada) Moss bags (45 days exp.)	Stereomicroscopy Hot needle test	Urban intensity:		[17]
			High = 5.8–15.0	MFs = 30–71%	
			Medium = 8.1–9.5	MFs = 25–43%	
			Low = 2.5–5.7	MFs = 55–69%	
				Length range = 0.03–4.51	
*Hypnum cupressiforme*	Semi-natural/rural sites (southern Italy) Field sampling	Stereomicroscopy FT-IR	Mean = 71 ± 13	MFs = 99%, Length range = 0.2–10.0	[30]
*Hypnum cupressiforme*	Parking area, urban roof and a rural area (southern Italy) Moss bags (6 weeks exp.)	Stereomicroscopy Raman spectroscopy	Range = 21–152 Mean = 102 ± 24	MFs = 99%, Length range = 0.2–10.1	[18]
*Pseudoscleropodium purum*	5 remote sites (central Italy) Field sampling	Stereomicroscopy Hot needle test	Range of mean values 12.5–21.2	MFs = 90%, Length range = 0.14–4.08 Fragments = 10%, Length range = 0.29–0.59	[31]
*Grimmia crinita*	Altitudinal transect (SW Iran) Field sampling	Stereomicroscopy Raman spectroscopy	Range = 0.1–1.55	MFs = 67%, Length range: from <0.1 (43%) to >1 (17%)	[32]
8 species, mostly *Hypnum cupressiforme*	Rural sites (Tuscany, Italy) Field sampling	Stereomicroscopy Hot needle test FT-IR	Range = 1.33–11.56 Mean = 5.28 ± 2.90	MFs = 86.8%, Length range = 0.068–4.932 Mean = 1.115–1.131	[33]
Unspecified	Industrial area (Kosovo) Field sampling	Stereomicroscopy	Range = 10–20	Length range = 0.25–3.00	[34]

## Data Availability

No new data were created or analyzed in this study. Data sharing is not applicable to this article.

## Abbreviations

The following abbreviations are used in this manuscript:

GPIB	Global Plastic Ingestion Bioindicators
LRTAP	Long-Range Transboundary Air Pollution
MFs	Microfibers
MPs	Microplastics
NPs	Nanoplastics
